# Thyroid Autoantibodies in the Cerebrospinal Fluid of Subjects with and without Thyroid Disease: Implications for Hashimoto's Encephalopathy

**DOI:** 10.1155/2015/819072

**Published:** 2015-12-21

**Authors:** Ioannis Ilias, Vasiliki Karagiorga, George Paraskevas, Anastasia Bougea, Mara Bourbouli, Athina Pappa, Stamatina Nikopoulou, Elisabeth Kapaki

**Affiliations:** ^1^Endocrine Unit, Elena Venizelou Hospital, 2 E. Venizelou Square, 11521 Athens, Greece; ^2^Neurology Department, University of Athens Medical School, Aeginition Hospital, 72-74 Vasilissis Sofias Avenue, 11528 Athens, Greece

## Abstract

*Introduction*. Plasma antithyroid peroxidase (anti-TPO) and anti-thyroglobulin antibodies (anti-Tg) are widely used in the diagnosis of autoimmune thyroiditis. No research has compared anti-TPO and anti-Tg both in plasma and cerebrospinal fluid (CSF) of healthy individuals vis-à-vis patients with thyroid disease.* Methods*. We measured anti-TPO and anti-Tg antibodies in plasma and CSF in nine subjects (mean age ± SD: 73 ± 6 years) with hypothyroidism and nine subjects (mean age ± SD: 73 ± 8 years) without thyroid disease.* Results*. The concentration of anti-TPO autoantibodies in CSF was very low compared to plasma in both subjects with thyroid and without thyroid disease (*P* = 0.007). CSF anti-Tg autoantibodies titers were very low compared to the plasma in subjects with thyroid disease (*P* = 0.004), whereas, in subjects without thyroid disease, this difference did not reach statistical significance (*P* = 0.063).* Conclusions*. Thyroid autoantibodies levels were low in plasma and CSF; we did not observe any transfer of thyroid autoantibodies from the peripheral blood to the CSF. Therefore, regarding Hashimoto's encephalopathy, where elevated antithyroid autoantibodies are often measured in blood, it is more likely that thyroiditis and encephalopathy represent nonspecific, but distinct, events of an aggressive immune system.

## 1. Introduction

The appearance of thyroid autoantibodies (in particular, antithyroid peroxidase; anti-TPO) in plasma and cerebrospinal fluid (CSF) is a necessary condition for the diagnosis of Hashimoto's encephalopathy (HE), a rare disease associated with autoimmune thyroiditis [1, 2]. The plasma level of antithyroid antibodies, however, is not related to the severity of the disease [3]. Thyroid autoantibodies in plasma are very common in the general population (10%–25% have them, particularly women) [3, 4] and one could speculate the transfer of autoantibodies from blood to the CSF. Corroborating the latter, anti-thyroglobulin (anti-Tg) antibodies were described in the CSF of patients with hypothyroidism in the past [5]. No research has evaluated simultaneously thyroid autoantibodies both in plasma and cerebrospinal fluid (CSF), in patients with thyroid disease and healthy individuals. Thus, the aim of this study was to search for anti-TPO and anti-Tg antibodies in CSF and assess them vis-à-vis their plasma concentrations in subjects with and without thyroid disease.

## 2. Subjects and Methods

We studied nine subjects without thyroid disease (2 men/7 women, mean age ± SD: 73 ± 6 years) and 9 subjects with hypothyroidism (one man/8 women, mean age ± SD: 73 ± 8 years), who were scheduled to undergo elective hip surgery. We excluded subjects with a history of autoimmune disease such as megaloblastic anemia, systemic lupus, recent use of iodinated contrast media, rheumatoid arthritis (RA), type 1 diabetes, HE, pregnancy, chronic kidney or liver diseases, or cancer. The study was approved by the Ethics Committee of Aeginition Hospital, University of Athens Medical School; in all subjects, written informed consent was obtained. Detailed medical history was obtained from all participants; all underwent a physical examination (and had a thorough neurological examination).

In all participants, morning blood samples were obtained. Lumbar puncture was performed at the L4-S1 intervertebral space by an experienced clinician, between 9 and 12 AM after overnight fasting for CSF sampling. The samples in polypropylene tubes were immediately centrifuged at 4°C, 2000 g, for 10 minutes and stored at −80°C. We measured anti-TPO and anti-Tg in plasma and CSF using electrochemiluminescence (Roche Cobas anti-TPO and anti-Tg assays on a Cobas e411 analyzer; Roche Diagnostics, Mannheim, Germany). The detection thresholds for anti-TPO and anti-Tg were 5 U/mL and 10 U/mL, respectively. Data of thyroid parameters were nonnormally distributed. Accordingly, nonparametric statistics (median, quartiles, and correlation according to Spearman) were used. The two*-*sample paired sign test was used for differences in antithyroid antibodies between the two groups. All statistical analyses were conducted using StataSE v.10 (StataCorp LP, Texas, USA; 2009); statistical significance was set to be at *P* < 0.05. For reference, anti-TPO and anti-Tg were also measured in blood and CSF samples from a 65-year-old woman with HE.

## 3. Results

**Figure 1:**
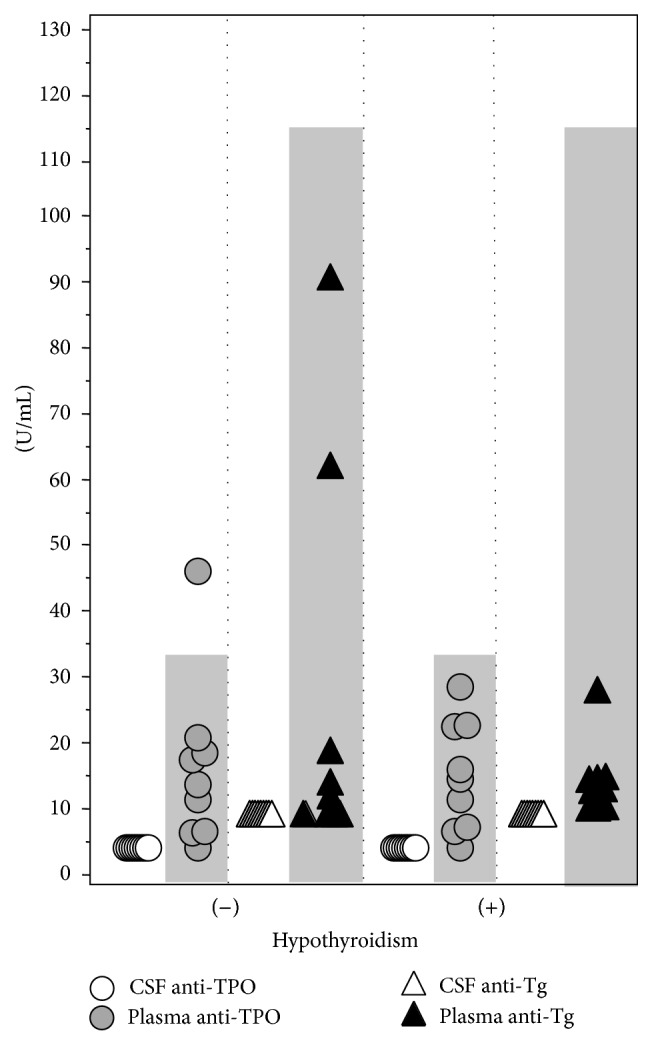
Autoantibodies levels in plasma and CSF; shaded gray areas denote plasma normal levels.

Medical history and physical examination from participants, as well as neuroimaging and CSF from the lumber puncture, were unremarkable. Baseline characteristics and thyroid autoantibodies were compared (Table 1). Neither gender nor age was significantly different among two groups. In the hypothyroid group, all patients were negative for both types of autoantibodies in plasma. In the control group, one subject was positive for plasma anti-TPO antibodies (47 U/mL). In both groups, CSF antithyroid antibodies were at the limit of detection. Thus, the concentration of TPO autoantibodies in CSF was very low compared to plasma in both subjects with thyroid and without thyroid disease (*P* = 0.007) (Figure 1). The concentration of anti-Tg autoantibodies in CSF was very low compared to plasma in subjects with thyroid disease (*P* = 0.004 sign test), whereas in subjects without thyroid disease this difference did not reach statistical significance (*P* = 0.062). In the patient with HE plasma anti-TPO and anti-Tg were >1000 U/mL, whereas CSF anti-TPO antibodies were 5 U/mL (at the limit of detection) and anti-Tg antibodies were 23 U/mL.

## 4. Discussion

In this study, we found very low CSF antithyroid autoantibodies compared to plasma in both subjects with and without thyroid disease. In our sample, the absence of inflammation by CSF analysis and the absence of white matter lesions were consistent with integrity of the blood-brain barrier. Thus, no transfer of thyroid autoantibodies from the peripheral blood to the CSF was noted, in the absence of major inflammation. This is in contrast to an earlier published work [5], where antithyroid autoantibodies were found in plasma and CSF of patients with goiter, myxedema, thyroiditis, or neurosyphilis at a concentration ratio of 2 : 1 (nevertheless, we have to bear in mind the vastly different methodologies used to this purpose, as assays show much variability in sensitivity and in their normal reference range as well [4]).

Most patients with HE have high plasma antithyroid antibodies; the CSF in these patients has been reported to be thyroid autoantibodies positive [6] or negative [7]. The findings of our HE patient are congruent with the former. The pathogenetic role of CSF antithyroid autoantibodies is unclear, as noted by Ferracci et al., who found high CSF antithyroid antibodies in six patients with HE, but they found negative results in 21 controls [6]. Although the patients had high plasma levels of antibodies, the anti-TG-TPO index was suggestive of intrathecal synthesis of these autoantibodies. The authors suggested that CSF antithyroid antibodies were a reliable marker to distinguish HE from other encephalopathies of unknown origin and that CSF autoantibodies levels should be studied regardless of negative plasma antibodies in patients with a high suspicion of HE [6].

It is questionable whether these detectable antibodies in the central nervous system are the culprit for HE, as no common antigen has been identified between the thyroid gland and the brain [8] despite the reported interaction of these autoantibodies with central nervous system tissues, forming immune complexes [9]. Interestingly, despite the prevailing view that features of severe vasculitis may be absent in HE [10, 11], there are some biopsy or autopsy studies that point to the contrary (with concomitant microcirculation disturbance) [12–14]; furthermore passive transfer of other encephalopathy-related antibodies in the brain has been shown [15].

This study has limitations: the small number of participants (but the nature of the assessment precluded a larger sample) and selection bias that cannot be ruled out, despite the implementation of strict exclusion criteria.

In conclusion, we did not observe the transfer of thyroid autoantibodies from the peripheral blood to the CSF. Regarding HE (where elevated antithyroid autoantibodies are often measured in plasma), the absence of transfer of thyroid autoantibodies from blood to CSF in this study lends credence to the fact that thyroiditis and encephalopathy represent nonspecific, but distinct, events of a too aggressive immune system, with no apparent causality [8]. As previously postulated [16], the presence of CSF anti-TPO and anti-Tg may only be a sign of a predisposition to produce multiple autoantibodies.

## Figures and Tables

**Table 1:** Comparison of baseline characteristics and thyroid autoantibodies between two groups; data are given as mean (SD) or median (interquartile range); *data from a patient with Hashimoto's encephalopathy (HE) are also given for comparison*.

	Patients with hypothyroidism (*n* = 9)	Controls (*n* = 9)	*P* value	Patient with HE (*n* = 1)
Gender (M/F)	1/8	2/9		0/1
Age (mean age ± SD); years	73 ± 8 years	73 ± 6 years	0.653	65
Anti TPO plasma; U/mL	15 (8–23)	15 (7–21)	0.453	>1000
Anti-Tg plasma; U/mL	14 (11–16)	13 (10–41)	0.207	>1000
Anti-TPO CSF; U/mL	5 (5-5)	5 (5-5)		5
Anti-Tg CSF; U/mL	10 (10-10)	10 (10-10)		23
